# Comparison of Pregnancies between Perinatally and Sexually HIV-Infected Women: An Observational Study at an Urban Hospital

**DOI:** 10.1155/2013/301763

**Published:** 2013-09-09

**Authors:** Martina L. Badell, Alisa Kachikis, Lisa B. Haddad, Minh Ly Nguyen, Michael Lindsay

**Affiliations:** ^1^Department of Gynecology and Obstetrics, Emory University School of Medicine, 69 Jesse Hill Jr. Drive, Atlanta, GA 30303, USA; ^2^Department of Medicine, Division of Infectious Disease, Emory University School of Medicine, Atlanta, GA 30303, USA

## Abstract

As perinatally HIV-infected (PHIV) women reach reproductive age, there is an increasing number who become pregnant. This is a retrospective cohort study of HIV-infected women who delivered from June 2007 to July 2012 at our institution. Maternal demographics, HIV characteristics, and obstetric and neonatal outcomes were compared. 20 PHIV and 80 SHIV pregnancies were reviewed. The groups had similar CD4+ counts, prevalence of AIDS, and use of antiretrovirals (ARV) at initiation of obstetrical care. PHIV women were significantly more likely to be younger, have a detectable viral load (35% versus 74%, *P* < 0.01), and have HIV-genotype resistance (40% versus 12%, *P* < 0.01) than the SHIV women. The median gestational age at delivery (38 weeks) and rates of obstetrical and neonatal complications were similar between the groups. While the overall rate of cesarean delivery (CD) was similar, the rates for CD due to HIV were higher in the PHIV group (64% versus 22%, *P* < 0.01). There was one case (5.3%) of mother-to-child transmission in the PHIV group versus two cases (2.6%) in the SHIV group. In our population, PHIV pregnant women have a higher rate of HIV-genotype resistance and higher rate of detectable viral load leading to a higher rate of CD secondary to HIV.

## 1. Introduction

The first cases of mother-to-infant or vertical transmission of human immunodeficiency virus (HIV) were described more than two decades ago. In the early 1980s, the majority of perinatally acquired HIV (PHIV) children did not survive beyond childhood. As the perinatally infected cohorts have benefitted from combined highly active antiretroviral therapy (HAART), they are living longer and the first wave is reaching adolescence and young adulthood. The estimated number of perinatally infected persons living with HIV was 6,051 in 2005 for the 33 jurisdictions with HIV being reported in the United States [1]. As perinatally HIV-infected females become sexually active, they are in turn at risk of transmitting HIV to their children. Currently health care providers of PHIV women are encountering reproductive health concerns in this population with little evidence to guide them.

Although HAART has markedly decreased the risk of perinatal HIV transmission in the USA among adult females infected with HIV and there is substantial literature regarding pregnancy outcomes in HIV-infected women, little is known about the course of pregnancy among perinatally infected females. In an editorial response to a report describing pregnancy in perinatally HIV-infected adolescents and young adults, the Centers for Disease Control (CDC) in 2003 recommended enhanced efforts to identify pregnancies among perinatally HIV-infected adolescents/young adults and more in-depth investigation of such pregnancies to better characterize the factors associated with these pregnancies and their outcomes [2]. Since 1998, 13 reports of pregnancies among perinatally infected adolescents have been described [2–14].

As the generation of perinatally HIV-infected women (PHIV) reaches reproductive age, there are an increasing number of them who will become pregnant. Given the current paucity of data regarding whether pregnancy and neonatal outcomes in women with perinatally infected-HIV are similar to sexually infected HIV women (SHIV), the objective of this study was to determine and compare the outcomes in these populations at our institution. 

## 2. Methods

A retrospective cohort study was performed of all PHIV-positive women who delivered at Grady Memorial Hospital beginning in June 2007 through August 2012. Grady Memorial Hospital is an inner-city hospital which cares for Medicaid, underinsured, and uninsured patients throughout the metro Atlanta area. This study received approval from Institutional Review Boards at both Emory University and The Grady Memorial Hospital Research Oversight Committee (IRB number 00051101). HIV-infected women were considered perinatally infected if they had a documented positive HIV test in infancy or childhood or if the patient's family history suggested perinatal infection. Each pregnancy of a PHIV woman was counted as a separate occurrence. A cohort of sexually infected HIV-positive women from our institution who delivered within three months of the specific pregnancy occurrence of a perinatally infected HIV-positive woman were chosen for comparison. Women were excluded if their pregnancy resulted in a spontaneous or therapeutic abortion at less than 20 weeks and if they did not deliver at our institution, had a multiple gestation, or were not diagnosed with HIV/AIDS prior to the index pregnancy. 

Demographic information regarding the patient's age at time of delivery, living environment, marital status, ethnic background, employment status, and education was collected as well as basic health information such as gravidity and parity, comorbidities, history of sexually transmitted infections or abnormal pap smears, and tobacco, alcohol, or illicit drug use. Maternal HIV characteristics included whether the patient met diagnostic criteria for AIDS and had been on HAART medication prior to pregnancy. In addition CD4 count and viral load at initiation of prenatal care were collected as well as presence of genotype resistance and undetectable viral load during the pregnancy. Obstetrical variables included estimated gestational age at initiation of prenatal care, number of prenatal clinic visits, and antepartum complications including antepartum admissions, preterm labor, preterm premature rupture of membranes, chronic hypertension, gestational hypertension and preeclampsia, gestational diabetes, and STIs diagnosed during pregnancy. Birth outcome variables included estimated gestational age at delivery, mode of delivery, birth weight, 1- and 5-minute apgar scores, neonatal ICU admissions, intrapartum or postpartum complications including postpartum hemorrhage, endometritis, or other infections, postpartum clinic visits, continuation of HAART therapy, postpartum contraception choice, and whether perinatal transmission of HIV to the neonate occurred. Data was analyzed using SPSS-15 (SPSS Inc., Chicago, IL, USA). Chi-square analysis, Student *t*-tests and Mann-Whitney *U* test were used when appropriate. An alpha level of less than 0.05 was used to determine significance. 

## 3. Results

During the study period, from June 2007 to July 2012, 20 PHIV women delivered at our institution. For comparison, data was collected on 80 SHIV women who delivered during this same time period. Maternal characteristics of the two groups are presented in Table 1. The majority of women in both groups were African American (95.0% PHIV and 87.5% SHIV). The PHIV group was significantly younger than the SHIV group (20.1 versus 28.9 years, *P* ≤ 0.5). The number of women on HAART therapy prior to pregnancy was comparable (47.4% PHIV versus 44.3% SHIV). The initial median CD4 count and viral load did not differ between the groups; however, the PHIV group had a significantly higher rate of genotype resistance (40% versus 12.5%, *P* ≤ 0.01) and detectable viral load during the pregnancy (65.0% versus 37.2%, *P* ≤ 0.01) than the SHIV group. 

The obstetrical characteristics of our cohorts are presented in Table 2. The PHIV group were significantly more likely to be having their first baby (60.0% versus 11.2%, *P* < 0.01), whereas the SHIV group were more likely than PHIV group to have had five or more pregnancies (33.8% versus 0%, *P* < 0.01). The median gestational age at the time of first prenatal visit and gestational age at delivery were comparable between the groups. A full-term delivery occurred in 95% among PHIV group compared to 85% among SHIV group, *P* = NS. The majority of parturients in both groups underwent cesarean delivery (CD) (55.0% PHIV and 56.2% SHIV). However, the rate of CD for an HIV-related reason was significantly higher in the PHIV group than in SHIV group (63.6% versus 22.2%, *P* ≤ 0.5). The median birth weight between the groups was comparable. 

The maternal and neonatal complication rates are outlined in Table 3. Overall the incidences of major complications were low and comparable between the groups. There was no difference in rates of preeclampsia, preterm birth, chorioamnionitis, or low birth weight. Both groups had a fairly high rate of antepartum admissions during the pregnancy (35.0% PHIV versus 25.0% SHIV). Also 20% of the perinatally infected group had a postpartum infection versus 8.8% in the sexually infected group (*P* = 0.22). In the perinatally infected versus sexually infected groups, the rates of low birth weight were 15.0% versus 12.8%, respectively, and the rates of NICU admission were comparable. There were no cases of maternal or neonatal mortality in either group. 

There was one case of mother-to-child transmission (MTCT) in the perinatally infected group (5.3%) versus two cases in the sexually infected group (2.6%). The case of MTCT in the PHIV occurred in a patient not taking her prescribed HAART who initiated prenatal care late at 18 weeks with a CD4 count of 13 and viral load of 24,500. She only came to a total of 5 visits and was not seen between 31 weeks and when she presented on labor and delivery with ruptured membranes at 40 weeks. During pregnancy her viral load was never undetectable and she only intermittently took her medications. One of the cases of MTCT in the sexually infected group occurred in a patient who received no prenatal care reportedly secondary to depression. She presented at term in labor not having taken any ART during the pregnancy with a viral load of 97,660 copies/mL. The other case in the sexually infected group occurred in a complaint patient who was on HAART prior to and throughout the pregnancy with viral load <1,000 copies/mL at the time of delivery. She received AZT during labor however for obstetrical indications due to arrest of fetal descent and non-reassuring fetal status she required an urgent CD. A large amount of blood was noted upon entry into the uterus concerning placental abruption and the baby was noted to be limp and pale and had swallowed a significant amount of blood requiring suctioning, intubation, and admission to the neonatal intensive care unit. Despite also receiving ART after birth, the baby was found to be HIV infected presumably from the ingestion of a large amount of maternal blood in utero.

## 4. Discussion

In this retrospective cohort study of 20 PHIV pregnant women compared to 80 SHIV pregnant women, we evaluated the demographics, HIV characteristics, and pregnancy outcomes among the two groups. Overall the groups were similar in regard to race, initial median CD4 count, prevalence of AIDS, and being on HAART at presentation. However, the women with perinatally infected HIV were younger and more likely to be having their first baby and had significantly higher rates of detectable viral loads with higher rates of HIV-genotype resistance. In regard to pregnancy outcomes, the groups were fairly similar in respect to gestational age at delivery, mode of delivery, and rates of obstetrical/neonatal complications; however, the perinatally infected group had significantly higher rates of CD due to detectable HIV viral load near the time of delivery. 

 Prior studies have also found that PHIV females appear to be at increased risk of delivering by CD. In the study by Williams et al. the rate of CD was 62%, with 75% of the cases being performed for HIV infection [12]. In a European study of PHIV females, 8 of the 9 (89%) were delivered by CD [10]. Compared to a vaginal delivery, CD is associated with increased morbidity including infection, excessive blood loss, postoperative pain, and need for repeat CD. Given the young age of these women and potential likelihood of future pregnancies, the optimal delivery would include viral suppression from HAART leading to opportunity for a vaginal birth.

 In the USA, guidelines treatment thresholds for nonpregnant HIV-infected women are the same as those for other HIV-infected adults and adolescents to decrease the risk of disease progression. Current adult treatment guidelines strongly recommend HAART for all individuals with CD4-cell counts <350 cells/mm^3^ based on randomized, controlled clinical trial data demonstrating a clear benefit in reduction of mortality and morbidity [15]. Overall, our PHIV and SHIV cohorts had a low rate of HAART usage with PHIV (47.4%) and SHIV (44.3%). Among the 13 PHIV with a diagnosis of AIDS or CD4 <350 at entry into OB care, only 5/13 (38%) were on HAART. Among the 41 SHIV with an AIDS diagnosis or CD4 <350 at OB entry, only 14/41 (34%) were on HAART. As this was a retrospective study, the exact reason for this low usage of HAART could not be elicited; however, based on review of the records most of the women in both groups who should have been on HAART but were not taking it were either noncompliant with a prescribed regimen or nonadherent to medical followup. The higher rate of HIV-genotypic resistance among PHIV is likely due to the fact that PHIV-infected women would be more heavily HAART experienced and thus would harbor more resistant HIV virus. However, adherence to antiretroviral therapy has previously been documented to be poorer during adolescence in HIV-infected individuals compared to younger children and adults [16] which places the PHIV population at higher risk of resistance. Poor compliance with medication and care significantly impacts virologic control at all ages. By focusing more attention and effort on this high-risk PHIV-infected patient population we may be able to achieve higher rates of compliance with medications and achieve lower viral loads. Ultimately higher rates of undetectable viral load could lead to lower rate of CD for HIV indications in this young population. 

The rate of mother-to-child transmission (MTCT) was 5.3% (1 case) in the PHIV group and 2.6% (2 cases) in the SHIV group. These rates are not statistically different given insufficient power due to the small number of subjects in each group; however, the absolute rates are higher than those desired in the United States today as when HIV is diagnosed before or during pregnancy, perinatal transmission can be reduced to less than 1% if appropriate medical treatment is given, the virus becomes undetectable, and breastfeeding is avoided [17]. The case in the perinatally infected patient was clearly secondary to noncompliance with medications and medical care as she was not taking her prescribed HAART, presented late for care (18 weeks), missed many prenatal appointments, and presented to labor and delivery more than 4 hours after rupture of membranes. One of the cases of MTCT in the sexually infected group was also secondary to medical noncompliance as she received no prenatal care and presented in active labor with a high viral load (97,660 copies/mL). The other case in the sexually infected group unfortunately occurred in a complaint patient who was on HAART prior to and throughout the pregnancy with viral load <1,000 copies/mL at the time of delivery and was thought to be secondary to fetal aspiration of maternal blood secondary to placental abruption. The rates of MTCT in prior studies evaluating PHIV women range from 10% (1/10) in the cohort evaluated by Williams et al. [12], 3.3% (1/32) in another American cohort [3], to 0% (0/9) in a European cohort [10]. Given the low numbers it is difficult to accurately predict or assess the true risk in the PHIV population. Our cases illustrate that compliance with ART treatment and following outlined prenatal care guidelines for all women with HIV are necessary to prevent MTCT; moreover that even under ideal circumstances it is unlikely that we will be able to entirely eliminate the risk of MTCT to 0%. 

Our study has several strengths. The number of PHIV pregnancies studied is fairly high given the relative rarity of this condition. By comparing the obstetrical and neonatal outcomes to sexually HIV-infected pregnant women we were able to identify the many ways in which their pregnancies are similar, and thus it is likely that prior data on HIV in pregnancy which is almost exclusively derived from sexually infected women is applicable to the perinatally infected women also. An additional strength is that the entire data was collected from a single institution and the sexually infected women were selected based on timing of delivery within 3 months of a perinatally infected woman. This should lead to similar baseline obstetrical and neonatal care received by both groups. 

The principal limitation of our study is its retrospective and observational nature. Given this, our results are descriptive and we were not able to detect any direct association. Further, as PHIV pregnancies are rare, we are underpowered to determine significant difference in many of the pregnancy-related outcomes. In the future, a larger multicenter study may help to determine any difference that may exist among these groups. Further research performed prospectively using methods to identify risk factors and reasons for noncompliance among the perinatally HIV-infected pregnant women may provide more insight into ways to lower viral loads, CD rates, and ultimately mother-to-child transmission rates. 

## 5. Conclusion

In our population, PHIV compared to SHIV parturients have a significantly higher rate of genotype resistance and higher rates of detectable viral load leading to a higher rate of CD for HIV-related reasons. Closer followup, monitoring and further education among PHIV women are warranted in an attempt to improve compliance with medications and care. In addition, all women of child bearing age should know their HIV status and be enrolled in care if positive. Reproductive healthcare education and preconception counseling would likely improve health outcomes in the PHIV-infected population; however integrating this into busy clinical practice is challenging. Planned and desired pregnancies in PHIV women on HAART at the time of conception with good adherence to medications and care throughout pregnancy should decrease HIV-related CD and ultimately repeat mother-to-child HIV transmission. 

## Figures and Tables

**Table 1:** Maternal characteristics of perinatally HIV-infected versus sexually HIV-infected parturients.

	Perinatally HIV-infected (*n* = 20)	Sexually HIV-infected (*n* = 80)	*P* value
Age (years)	20.1 ± 2.7	28.9 ± 5.8	**<0.05**
Race			NS
African American	19 (95.0%)	70 (87.5%)	
Others*	1 (5.0%)	10 (12.5%)	
Employed	12 (66.7%)	58 (76.3%)	NS
Insurance			NS
Medicaid	20 (100%)	54 (71.1%)	
Private	0 (0%)	4 (5.3%)	
Grant Ryan White	0 (0%)	18 (23.7%)	
Hx of sexually transmitted infection	17 (85%)	49 (61.3%)	NS
Current substance abuse	0 (0%)	13 (16.2%)	NS
AIDS	11 (55%)	30 (37.5%)	NS
HAART prior to pregnancy diagnosis	9 (47.4%)	33 (44.6%)	NS
HIV care at pregnancy diagnosis	14 (70%)	45 (56.2%)	NS
Initial CD4 count (cells/mm^3^)			
Median	322	393	NS
Range	9–1199	6–1215	NS
Initial viral load (copies/mL)			
Median	2060	655	NS
Range	0–71, 840	0–284, 640	NS
Genotype resistance	8 (40%)	10 (12.5%)	**<0.01**
Viral load undetectable during pregnancy	7 (35.0%)	59 (73.8%)	**<0.01**

NS: not significant.

*Others include Caucasian, Hispanic, and Asian.

**Table 2:** Obstetrical characteristics of perinatally HIV-infected versus sexually HIV-infected parturients.

	Perinatally HIV-infected (*n* = 20)	Sexually HIV-infected (*n* = 80)	*P* value
Gravidity			**<0.01**
1	12 (60%)	9 (11.2%)	
2–4	8 (40%)	44 (55%)	
5+	0 (0%)	27 (33.8%)	
Gestational age at first prenatal visit (weeks)			NS
Median	13.5	14	
Range	5–29	5–38	
Gestational age at delivery			NS
Median	38.2	38	
<24 weeks	1 (5%)	2 (2.5%)	
24–36.6 weeks	0 (0%)	10 (12.5%)	
>37 weeks	19 (95%)	68 (85%)	
Mode of delivery			NS
Vaginal	9 (45%)	34 (42.5%)	
Cesarean	11 (55%)	45 (56.2%)	
Dilation and evacuation	0 (0%)	1 (1.2%)	
Indication for cesarean delivery			**<0.05**
HIV related	7 (63.6)	10 (22.2%)	
Non-HIV related	4 (36.4%)	35 (77.8%)	
Birth weight (grams)			NS
Median	2872	2990	
Range	(240–3840)	(420–4110)	

**Table 3:** Maternal and neonatal complications of perinatally HIV-infected versus sexually HIV-infected parturients.

	Perinatally HIV-infected (*n* = 20)	Sexually HIV-infected (*n* = 80)	*P* value
Gestational HTN	1 (5%)	4 (5%)	NS
Preeclampsia	2 (10%)	3 (3.8%)	NS
Gestational diabetes	0 (0%)	1 (1.2%)	NS
PROM	0 (0%)	4 (5%)	NS
PTB	1 (5%)	11 (13.8%)	NS
APU admission	7 (35.0%)	20 (25.0%)	NS
Intrapartum hemorrhage	2 (10%)	6 (7.5%)	NS
Chorioamnionitis	0 (0%)	0 (0%)	NS
Postpartum infection	4 (20%)	7 (8.8%)	NS
LBW	3 (15.0%)	10 (12.8%)	NS
5 min APGAR < 7	1 (5.0%)	4 (5.1%)	NS
NICU admission	3 (15.0%)	13 (16.7%)	NS
Perinatal HIV transmission	1 (5.3%)	2 (2.6%)	NS
